# Emerging Threats of Highly Pathogenic Avian Influenza A (H5N1) in US Dairy Cattle: Understanding Cross-Species Transmission Dynamics in Mammalian Hosts

**DOI:** 10.3390/v16111703

**Published:** 2024-10-30

**Authors:** Chithra C. Sreenivasan, Feng Li, Dan Wang

**Affiliations:** Department of Veterinary Science, Maxwell H. Gluck Equine Research Center, University of Kentucky, Lexington, KY 40546, USA; c0sree03@louisville.edu

**Keywords:** high pathogenic avian influenza (HPAI), H5N1, cattle, transmission

## Abstract

The rapid geographic spread of the highly pathogenic avian influenza (HPAI) A(H5N1) virus in poultry, wild birds, and other mammalian hosts, including humans, raises significant health concerns globally. The recent emergence of HPAI A(H5N1) in agricultural animals such as cattle and goats indicates the ability of the virus to breach unconventional host interfaces, further expanding the host range. Among the four influenza types—A, B, C, and D, cattle are most susceptible to influenza D infection and serve as a reservoir for this seven-segmented influenza virus. It is generally thought that bovines are not hosts for other types of influenza viruses, including type A. However, this long-standing viewpoint has been challenged by the recent outbreaks of HPAI A(H5N1) in dairy cows in the United States. To date, HPAI A(H5N1) has spread into fourteen states, affecting 299 dairy herds and causing clinical symptoms such as reduced appetite, fever, and a sudden drop in milk production. Infected cows can also transmit the disease through raw milk. This review article describes the current epidemiological landscape of HPAI A(H5N1) in US dairy cows and its interspecies transmission events in other mammalian hosts reported across the globe. The review also discusses the viral determinants of tropism, host range, adaptative mutations of HPAI A(H5N1) in various mammalian hosts with natural and experimental infections, and vaccination strategies. Finally, it summarizes some immediate questions that need to be addressed for a better understanding of the infection biology, transmission, and immune response of HPAI A(H5N1) in bovines.

## 1. Introduction

Influenza viruses belong to the *Orthomyxoviridae* family, and there are four known types of influenza viruses: A, B, C, and D. Influenza A and B viruses can cause seasonal influenza, with influenza A virus (IAV) having the potential to trigger an influenza pandemic with high morbidity and mortality. Influenza type A viruses are mainly classified into subtypes based on the two surface glycoproteins: hemagglutinin and neuraminidase. Altogether, there are 18 hemagglutinin (HA) subtypes and 11 neuraminidase (NA) subtypes [1]. Aquatic waterfowls are considered natural reservoirs for IAV except for H17-18 and N10-11, which are bat influenza A-like viruses and are restricted to bats [2,3,4,5]. Since the 1900s, four pandemics have occurred: two caused by H1N1 viruses (1918, 2009), one by the H2N2 virus (1957), and one by the H3N2 virus (1968) [6]. These pandemic viruses are believed to have originated from birds or pigs and have acquired human adaptation mutations leading to sustainable human-to-human transmission. Although influenza C and D viruses do not cause pandemics, their risk to human health cannot be ignored. Influenza C virus (ICV) can cause mild illness in humans, but severe symptoms, such as pneumonia, bronchitis, and bronchiolitis, can develop in children under two years old [7,8]. Influenza D virus (IDV) uses cattle as the reservoir and can infect other animals, but its impact on human health is not fully understood [9,10,11]. Before the emergence of IDV in cattle, cattle were not recognized as a susceptible host for influenza viruses [12,13,14].

The avian influenza (AI) virus is type A. Based on the intravenous pathogenicity test performed on specific pathogen-free (SPF) chickens, AI viruses are divided into highly pathogenic avian influenza (HPAI) virus and low pathogenic avian influenza (LPAI) virus. To produce infectious virus particles, the HA precursor needs to be cleaved into HA1 and HA2 subunits by a host protease. The HPAI HA can be cleaved by furin-like proteases ubiquitously expressed in most cell types in the body, whereas the LPAI HA can only be cleaved by certain trypsin-like proteases from host cells in the respiratory and enteric tracts [15]. LPAI viruses can evolve into HPAI viruses through mutational changes in the proteolytic cleavage site. Several H5 and H7 subtypic conversion events have occurred in Europe, Asia, North America, South America, Africa, and Oceania [16]. The robust replication property allows HPAI viruses to replicate in multiple organs, causing significant fatalities and leading to economic loss to the poultry industry. HPAI viruses possess a polybasic hemagglutinin cleavage site (HACS) motif that LPAI viruses lack. This motif enables the HPAI virus to replicate productively in the vascular system and multiple organs in avian species, leading to highly contagious and severe infection [17]. Historically, LPAI viruses replicate in the epithelial cells of the respiratory and digestive tracts, and kidneys [17,18]. The removal of the polybasic site motif by reverse genetics decreased the virulence of HPAI A(H5N1) [19].

HPAI viruses have caused considerable death in commercial poultry, resulting in significant economic losses worldwide. HPAI H5 or H7 viruses infect chickens, normally with mortality rates of up to 90% to 100% [20]. Epidemiological and phylogenetic studies on previous epizootics have shown that LPAI viruses circulated more in wild and free-living aquatic populations, while HPAI was more geographically limited and primarily affected commercial gallinaceous species [21]. The HPAI A(H5N1) virus was initially isolated from a goose in the Guangdong Province, China in 1996. The prototype strain A/Goose/Guangdong/1/1996 (H5N1), designated as the Gs/Gd lineage, expanded geographically, causing widespread mortality in wild birds and poultry and fatal deaths in human populations [21,22,23].

According to the HA sequence differences, HPAI H5Nx viruses are divided into ten single-order clades and multiple subclades [24,25]. Among the different clades, the prolonged circulation of HPAI A(H5N1) clade 2.3.4.4b viruses globally and their rapid evolution through genetic reassortment with LPAI and other influenza strains facilitated adaptive changes in the HA specificity, affecting not only avian hosts but also other terrestrial and marine mammalian species worldwide [25]. In Europe, multiple genotypes of clade 2.3.4.4b viruses began circulating after two consecutive epidemic waves in 2020–2021 and 2021–2022, with the latter being the largest epidemic facilitating adaptive mutations and new ecological niches. Panzootic H5N1 clade 2.3.4.4b viruses evolved rapidly through frequent reassortments and have spread to Africa, Asia, Europe, North America, and South America via migratory birds, leading to substantial outbreaks in domestic poultry and wild birds [23]. In North America, Gs/Gd H5-lineage viruses were first reported in 2014 along the Pacific Flyway in waterfowls and subsequently in other wild birds such as snow geese and ring-necked ducks in the Mississippi Flyway [26]. Multiple outbreaks were reported in commercial and backyard poultry until 2016. The transatlantic spread of HPAI H5 clade 2.3.4.4b viruses to North America occurred through wild birds in Eastern Canada in December 2021, and the virus was closely related to clade 2.3.4.4b viruses identified in Europe in the Spring of 2021 [27]. Following this, the first outbreak in US commercial poultry was reported in January 2022 and has affected more than 90 million poultry in 48 states [28].

## 2. Recent Bovine Influenza A Virus Outbreak in Domestic Ruminants in the US

**Figure 1:**
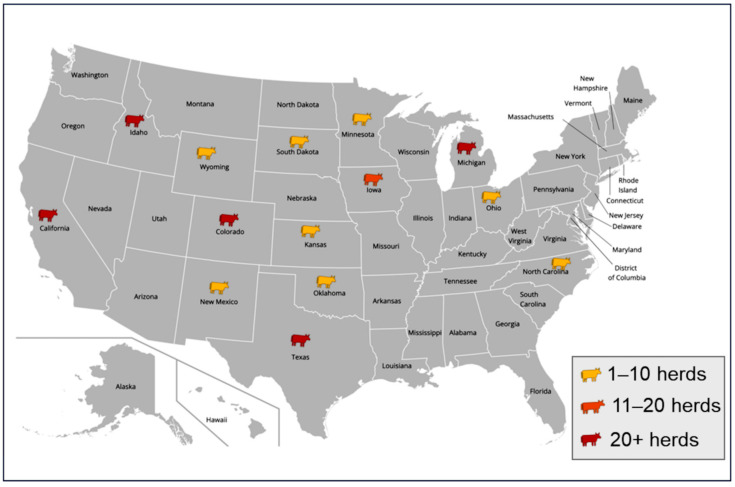
Current status of HPAI A(H5N1) virus spread in the US dairy cow herds. As of 14 October 2024, HPAI A(H5N1) virus was detected in 299 dairy cow herds in fourteen states of the US. The number of herds affected in each state was color-coded and shown. Data were obtained from USDA website (https://www.aphis.usda.gov/livestock-poultry-disease/avian/avian-influenza/hpai-detections/livestock, accessed on 15 August 2024). Map image was downloaded from https://inkpx.com, accessed on 15 August 2024.

In March 2024, juvenile goats with neurologic symptoms on an agriculture farm in Minnesota tested positive for the HPAI A(H5N1) virus, which marked the first case of HPAI A(H5N1) infection in domestic ruminants in the US [29]. The deceased goats were five to nine days old, and the HPAI A(H5N1) virus was detected in the brain, as well as in other tissues [30]. The poultry on the same farm was infected by the HPAI A(H5N1) virus in February, and the virus may have spilled over to the goats, as they shared space and water on the farm. HPAI A(H5N1) virus was also detected in sick cattle in Texas in March 2024. The initial genomic analysis of the virus did not detect known antiviral resistance markers or substitutions related to mammalian host adaptation signature mutations [31]. The HPAI A(H5N1) virus causing outbreaks in cattle belongs to the 2.3.4.4b clade genotype B3.13. As of October 2024, the H5N1 outbreaks were reported in 299 dairy cow herds across a total of fourteen states, in the US (Figure 1) [32]. HPAI A(H5N1) virus-infected cattle exhibited fever, lethargy, inappetence, gastrointestinal (GI) symptoms, and a significant drop in milk production. GI symptoms such as reduced rumination and changes in the fecal consistency, such as diarrhea or dry feces, were also reported. Furthermore, the HPAI A(H5N1) virus was detected in their raw milk at high concentrations, and the milk was also thickened or colostrum-like. HPAI A(H5N1) virus was also detected in alpacas, and the viral genome shared a sequence identity with the circulating B3.13 strain in dairy cattle [33].

Two unusual host infections of HPAI A(H5N1) in goats and cows in March 2024 highlighted that the spring migration of wild birds may have played an important role in the virus spread [34]. It has been suggested that dairy cows might have contracted the virus from wild birds, given that the dead birds were found on the same farm property. Potential transmission could occur directly or indirectly through fomites or by the environmental contamination of feed and water [35]. Recent developments also point towards the possibility of using poultry manure/litter in cattle feed as another potential source of infection in US states, as there are no strict federal regulations in the US regarding this practice (https://www.aces.edu/blog/topics/beef/feeding-broiler-litter-to-beef-cattle/, accessed on 29 July 2024). Although this is not scientifically established, it is considered a potential risk factor [36], and hence, the role of poultry litter in cattle feed cannot be dismissed and should be further investigated. It is still unclear how these viruses were transmitted from birds to domestic ruminants. HPAI A(H5N1) viruses can replicate in the epithelial cells of the gastrointestinal and respiratory tracts in birds, enabling transmission by both fecal-oral route and aerosols. Farm water and fomites may become contaminated by infected birds, which could be a plausible way for H5N1 virus transmission from birds to cattle. Alternatively, goats and cattle might be affected by an aerosolized virus. More studies are needed to address questions on virus transmission to cattle and goats from infected birds.

Influenza virus can cause gastrointestinal and respiratory illness in infected animals, but the goats and cattle demonstrated illness beyond that. The fact that goats showed neurologic symptoms and the virus detection in the brain suggests that the brain is a viral target, consistent with other mammalian host infections of HPAI A(H5N1) [37]. High levels of the H5N1 virus were detected in the raw milk of sick dairy cows, suggesting that the virus can replicate efficiently in dairy cow mammary glands. The clinical manifestations observed in both sick goats and cattle need more attention. Recently, Kristensen et al. reported that both human-type and avian-type sialic acid receptors are widely expressed in cattle mammary glands [38], which provides new insights into the HPAI A(H5N1) tropism in cow mammary glands.

Some earlier studies showed that dairy cows can be infected by H1N1 and H3N2 viruses in the late 1990s [39,40]. Affected dairy cows showed a significant drop in milk production. In addition to the reduction in milk yield, IAV-infected cattle also developed other clinical symptoms, such as fever, lack of appetite, and lethargy. Some infected dairy cows also manifested nasal discharge or respiratory noise. Gunning et al. performed HI assays to evaluate the prevalence of IAV infections in cattle. Of the 40 cattle investigated, 60% showed elevated antibody titers to the H1N1 virus, and 65% showed elevated antibody titers to the H3N2 virus [39]. Crawshaw et al. further demonstrated an association between elevated titers to H1N1 and H3N2 viruses and reduced milk yield in cattle [41]. In this regard, the H5N1 infection in cattle with a significant drop in milk yield that we observed is not entirely new.

HPAI A(H5N1) emerging in US dairy cattle has posed a significant health threat to dairy farm workers. High levels of H5N1 viruses were detected in raw milk, which may be transmitted to workers during handling of raw milk/milking devices. Additionally, infected cattle may shed the virus in feces contaminating the surrounding environment, further enhancing the health risk to dairy farm workers and other animals on the property. More importantly, the virus-contaminated milking machines and the environment may spread the virus to healthy cattle. In Japan, HPAI A(H5N1) and HxN1 were detected in 2.2% of the blow flies (*Calliphora nigribarbis*) collected, with higher prevalence near areas concentrated with HPAI-infected cranes. These flies feed on decayed birds, animal carcasses, and excreta. Blowflies will not support virus replication; however, the ingested virus can remain infective for 48 h. In Japan, blow fly migration to the HPAI endemic lowland areas in early winter suggests that these flies could be potential vectors of infection by contaminating surfaces, feed, and water in the farm premises [42]. Further studies are needed to analyze such potential links in the recent bovine H5N1 outbreaks in the US. Cattle transportation may facilitate further spread of the virus to other herds and mammalian hosts. Currently, the tested samples are limited in size, and there is concern that the H5N1 virus may spread in cattle more widely than initially thought, as the virus was detected in a lung sample of an asymptomatic cow. To date, there is no information available about infections in beef cattle and other cattle species, which requires immediate attention. 

Historically, influenza viruses have jumped species barriers facilitating efficient and sustained cross-species transmission at the avian–mammalian host interface [43,44,45,46]. The vast genetic diversity of influenza viruses, due to their quasispecies structure, adaptation, and persistence, enables them to escape the immune selection pressure and widen their host range into new avian and mammalian hosts. Among the known classical influenza A subtypes, H1-16 and NA 1-9 have been detected in wild aquatic birds, including ducks, geese (order Anseriformes), and gulls (order Charadriiformes). Some HA subtypes are species-specific, such as H13 and H16, which are predominantly seen in gulls. The H5 clade 2.3.4.4b is known for its high plasticity and has recently established itself in various mammalian species as its intermediate hosts.

**Figure 2:**
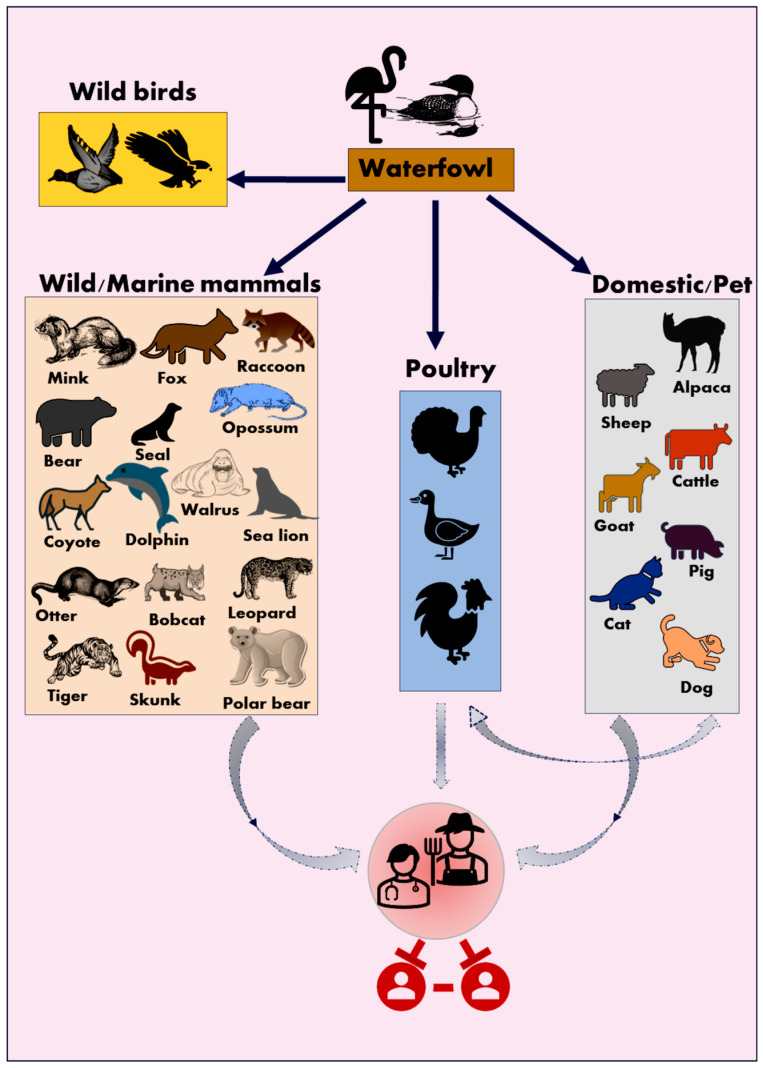
Cross-species transmission and host range of HPAI A(H5N1). Wild aquatic birds are the reservoir hosts for type A avian influenza viruses. Wild birds, wild mammals, poultry, and domestic/pet animals were affected by HPAI A(H5N1). The bold dark blue arrows denote frequent spillover events, while the gray arrows denote sporadic/infrequent spillover. Sustained human-to-human transmission has not been reported. Vector images were downloaded from Freepik (www.freepik.com, accessed on 15 August 2024), vecteezy (https://www.vecteezy.com/, accessed on 15 October 2024), and Microsoft Office icons.

The panzootic HPAI A(H5N1) virus can infect wild birds and commercial poultry, as well as a wide range of wild, marine, and domestic mammal species, including sea lions, dolphins, foxes, bobcats, Virginia opossums, raccoons, coyotes, Prairie voles, American martens, minks, squirrels, striped skunks, seals, black bears, polar bears and tigers (Figure 2) [43,47,48,49,50,51,52].

### 2.1. Natural Infection of HPAI A(H5N1) in Wild Mammals

The spillover events of the HPAI A(H5N1) virus in wild mammals occur through direct contact, feeding on virus-infected birds, or by the contamination of premises, feed, and water by bird excreta, saliva, or nasal secretions. HPAI A(H5N1) infections in wild mammals can be fatal [53,54]. In 2003, two tigers and two leopards in a zoo in Thailand were reported to be infected by the HPAI A(H5N1) virus after eating raw chicken that was possibly infected with virus. These zoo animals showed high body temperature, respiratory distress, increased nasal discharge, severe thrombocytopenia, leukopenia, elevated hepatic enzymes, and neurologic manifestations before death [49,55]. The duration of the disease was three days. Another fatal H5N1 outbreak in tigers in mid-October 2004 in Thailand also revealed that successful horizontal transmission occurred in tigers. The H5N1 isolates of tiger origin were phylogenetically similar to the chicken H5N1 and demonstrated the classical unique E627K substitution of PB2, a remarkable adaptive mutation facilitating host tropism in different mammals and 5 codon deletion in NS [55]. Another substitution, PB2-D701N was detected in the virus from a dead lynx in Finland and was also associated with enhanced viral replication in mammals [56]. In China, fatal H5N1 infection occurred in zoo-housed tigers and peacocks in 2014–2015, and the phylogenetic analyses of the virus isolates from tiger and peacock demonstrated the closest homology to reassortant avian influenza viruses of 2.3.4.4e and 2.3.2.1b subclades [57].

During the initial outbreaks of HPAI A(H5N1) in 2021–2022, the first detection of the Eurasian strain in a wild mammal was detected in red foxes and bobcats in the US in May 2022 [53,54]. Although the seroprevalence of influenza subtypes, H10N7, H4N6, H4N2, H3, and H1 were earlier reported in wild raccoons in 2008 [57,58], it was found that raccoons are naturally susceptible to the Eurasian (EA) lineage of HPAI A(H5N1) Gs/Gd 2.3.4.4b and its reassortants with North American (AM) lineage avian influenza viruses (EA/AM). Raccoons also possess both avian and human-type sialic acid receptors with a distribution pattern similar to the human respiratory tract, making them a suitable ecological niche for both avian and human influenza viruses [58]. Similarly, HPAI A(H5N1) of the EA and EA/AM lineages caused fatal infections in mountain lions in six different states of the US during the outbreaks from 2022–2024 [54]. HPAI A(H5N1) primarily affected the mountain lions’ lungs and brain, causing encephalitis and pulmonary edema [59]. H5N1 infections have been reported in various bear species such as brown bears, American and Asian black bears, grizzly bears, and Kodiak brown bears [54]. Free-range black bears in Quebec, Canada showed neurological signs after H5N1 infection, with histopathological changes in the brain characterized by neuronal necrosis and inflammatory and degenerative changes in the meninges and cerebrum. Phylogenetic analyses revealed the close homology of HA gene to Eurasian HPAI A(H5N1) clade 2.3.4.4b, sharing more sequence identity to sequences from red foxes than minks or skunks. The whole genome of the HPAI A(H5N1) of black bear origin also shared similarities to the Newfoundland-like H5N1 viruses, which likely came by transatlantic route from Europe to Canada, as the habitat of these bears was located close to the Atlantic Americas avian flyway [50].

In 2022, minks on a farm in Spain tested positive for the HPAI A(H5N1) virus. Neurological signs such as tremors and ataxia were reported, along with inappetence, hypersalivation, bleeding from the snout, and depression. Further examination of the infected minks revealed lung lesions [60]. In 2024, the HPAI A(H5N1) clade 2.3.4.4b virus was detected in minks US [54]. Minks are susceptible to several different IAV subtypes, suggesting the possibility of the mink species as an intermediate host, facilitating interspecies transmission between birds and mammals including humans [61]. In 2023, fur farms in Finland reported fatal HPAI A(H5N1) infection in American minks, arctic foxes, red foxes, and their crossbreeds, as well as sables and raccoon dogs, mostly contracted from the wild birds in the farm premises [62]. In Finland, an otter was found dead due to HPAI A(H5N1) virus infection, and a microscopic examination showed meningoencephalitis in the brain. The substitution PB2-E627K was also detected in the H5N1-infected otter and red fox. PB2-E627K was reported as the main determinant of virulence in the H5N1 virus [63]. Foxes infected after eating HPAI A(H5N1) virus-infected birds exhibited respiratory and neurologic symptoms [46,64].

### 2.2. Natural Infection of HPAI A(H5N1) in Marine Mammals

Cetaceans and pinnipeds were also affected by the HPAI A(H5N1) clade 2.3.4.4b concomitant with the spread of HPAI viruses in carnivores and mesocarnivores. Similar to wild mammals, neurological involvement was more prevalent than respiratory signs. In 2022, dolphins and sea lions in Peru tested positive for HPAI A(H5N1), and these animals were either dead or showed respiratory symptoms and/or neurological signs [51,65]. The PB2-D701N mutation was also detected in the H5N1-infected sea lion in Peru [65]. In June 2022, unusual mortality events occurred in stranded harbor and grey seals along the North Atlantic coast of the US. Swab samples collected from these dead animals tested positive for HPAI A(H5N1). Transmission from birds to seals might have occurred through environmental contamination. Some of these seals exhibited respiratory signs along with neurological symptoms [51]. Whole genome sequencing analyses of seal- and avian-derived sequences from New England demonstrated 4 shared and 37 seal-acquired amino acid substitutions in different viral proteins in the second wave of outbreaks compared to the first wave of HPAI infections [51]. Around late June 2022, clade 2.3.4.4b H5N1 virus spilled from wild birds to harbor porpoises in Sweden. The clinical manifestations were similar to the pinnipeds, characterized by circling, disorientation, and terminal drowning. The major lesion observed was meningoencephalitis and purpoise-origin H5N1 clustered closely with avian H5N1 from the same location and time frame. Interestingly, there were no mutations in the genome, which implies that these viruses can spill over and sustain transmission to mammalian hosts in the absence of adaptive mutations [66]. In 2022, H5N1 also affected bottlenose dolphin in Florida with a higher viral load in brain than in the lungs. The main CNS lesions included marked mononuclear cell infiltrations in the brain and meninges, neuronal necrosis, and neuropil malacia [48]. Whole genome sequencing and further phylogenetic analyses revealed that the dolphin-derived virus is an EA/AM lineage reassortant genotype B1.2 and showed 99% sequence identity to the H5N1 sequences circulated across the Atlantic and Mississippi Flyways in the spring of 2022 but was not related to the unreassorted seal H5N1 genotype A2, suggesting the absence of transmission from seals [48]. Natural infections of polar bears were also reported in Alaska in 2023 with major lesions, including skin lesions, congestion in the liver and lungs, pericardial effusion, congestion and inflammatory changes in the brain, and empty stomach. Histopathological findings included vasculitis and rarefaction, pulmonary edema, pneumonia, and multifocal ulcerative dermatitis [67]. The polar bears also exhibited some neuropathological lesions such as granulocytic and mononuclear meningoencephalitis with microgliosis, neuronal necrosis, neuronophagia, vasculitis, and parenchymal rarefaction [67].

### 2.3. Natural Infection of HPAI A(H5N1) in Domestic/Companion Animals

As discussed above, the goats infected by HPAI A(H5N1) virus also demonstrated neurologic symptoms [29,68]. This study, together with published work, indicate that the HPAI A(H5N1) virus can be neurotropic to mammals and cause a disorder in the central nervous system (CNS). Cats can also be infected with H5N1 2.3.4.4b, and the clinical signs are conjunctivitis, periocular swelling, depression, lethargy, respiratory distress, oculonasal discharge, and neurological signs such as circling and blindness. Since the recent emergence of H5N1 in bovines, farm and domestic cats have been increasingly affected, and so far, 21 domestic cats have been reported positive [34]. Fatality has been reported in domestic cats, and the transmission is mainly through feeding on colostrum or milk from H5N1-positive cows [34].

Similar to wild mammals, cats and dogs can be infected by HPAI A(H5N1) virus, and infections can be fatal in some cases [69,70,71]. In June 2023, cats in Poland tested positive for HPAI A(H5N1) virus, with the possible infection route being contaminated cat food [72]. Cats infected by HPAI A(H5N1) virus have also been reported in Germany, the US, and South Korea [73,74,75]. In April 2024, cats on an H5N1-positive dairy cow farm in the US were also infected with HPAI A(H5N1) virus [34,68]. The infection in these cats might have caused due to consuming unpasteurized milk from virus-infected dairy cows. The virus infection is fatal to cats, and the deceased cats from a Texas dairy farm showed signs of meningoencephalitis, pneumonia, myocarditis, and chorioretinitis [34]. An experimental study on cats demonstrated that they can be infected with the HPAI A(H5N1) virus by intratracheal inoculation or by consuming virus-infected chickens [76]. The study also found that virus-infected cats can transmit the virus to other cats. Furthermore, the HPAI A(H5N1) virus can also infect dogs. In 2023, a dog in Canada was confirmed to be infected by HPAI A(H5N1) after chewing on a wild goose [77]. The infection in this dog was fatal, and the necropsy demonstrated severe respiratory pathology. In Italy, an HPAI A(H5N1) outbreak on a poultry farm also affected a domestic cat and five dogs on the farm, although both cat and dogs remained asymptomatic [69,78]. Taken together, these clinical cases and studies emphasize that consuming raw meat from dead birds or the fecal contamination of feed or water can be an important source of cat and dog infections. These infections in companion animals pose a potential health risk to their owners and veterinarians.

### 2.4. Experimental Infection of HPAI A(H5N1) in Mammalian Hosts

The experimental infection of yearling heifers with A/dairy cattle/Texas/24-008749-002/2024 inoculated via respiratory route through aerosols induced no overt clinical signs other than the presence of nasal secretion. However, all the heifers were infected with seroconversion, as evidenced by virus detection in the ocular, oropharyngeal, and nasal tissues, as well as seroconversion [79]. Interestingly, the intramammary inoculation of the same virus in three-year-old, non-pregnant, lactating Holstein cows at 280 days of their primary lactation, replicated symptoms associated with field conditions such as lethargy, inappetence, decreased rumen motility, diarrhea or dry feces, mastitis, and changes in the milk consistency and color in 24-day study period [79]. The decrease in rumen motility was noticed after 1-day post-infection (dpi) and lasted for 7 days. Milk production declined from 1–4 dpi, continuing through 10–12 dpi, with an overall milk reduction of 71–77% in the lactating group. Histopathological analyses also demonstrated that the inflammatory changes were mostly present in the mammary glands, with only minimal pulmonary consolidative changes in both age groups. Neurological signs or lesions were absent in either of the age groups [79]. A cat-derived H5N1 isolate, A/cat/Germany/R606/2006 (H5N1), did not replicate efficiently in calves afterhigh-dose intranasal inoculation. Only a few animals demonstrated nasal shedding, and the animals did not show any clinical signs. Interestingly, all the calves seroconverted [80].

Another study using A/Cattle/Texas/063224-24-1/2024 inoculated through the oronasal route in calves also caused mild to moderate respiratory infection with nasal secretions and coughing but did not transmit disease by contact [81]. However, lactating cows (multiparous with ages between 4 and 8 years, 12 months post last calving) infected via the intramammary route were severely affected, exhibiting fever, lethargy, postural and motion disorders, respiratory distress, anorexia, dehydration, and mastitis. The onset of severe mastitis started from 2 dpi, and a 90% decline in milk production was noticed. Overall, the deteriorated body condition and postural disorders called for early euthanasia in some of the lactating cows [81]. Similar to the other study, the mammary glands showed more pronounced lesions; however, pulmonary congestion, edema, hemorrhage, and pleural adhesions were also noted. The age and comorbidities of the multiparous lactating cows contributed to the disease severity, leading to euthanasia prior to the set time points [81].

To assess the transmissibility of the HPAI bovine H5N1 through the oral consumption of milk, BALB/C mice were orally inoculated with milk from the infected cows and compared to mice inoculated with milk from the healthy cows. The high-dose infected mice showed substantial weight loss and death, whereas the disease manifestation and virus replication were inapparent in low-dose groups [82]. Virus dissemination to respiratory (nasal turbinates, lung) and non-respiratory organs (brain) was observed in high-dose groups. Furthermore, the intranasal inoculation of A/dairy cattle/New Mexico/A240920343-93/2024 (bovine H5N1), A/dairy cattle/New Mexico/A240920343-93/2024 (human H5N1) and (A/Isumi/UT-KK001-01/2018 (H1N1) in female BALB/C mice caused systemic infections infecting all respiratory and non-respiratory organs, including mammary glands and muscles, in both H5N1 groups, whereas H1N1 replication was confined to the respiratory tissues [82]. Disease transmission was also observed from lactating, intranasally inoculated female mice to their pups [82].

The experimental infection of the HPAI A(H5N1) clade 2.3.4.4b isolate, A/chicken/Germany/AI04286/2022 in 4-month-old pigs via intranasal and oral routes yielded marginal viral replication in respiratory and intestinal tissues and seroconversion in 1 out of 8 animals (intranasally inoculated). No clinical signs or pathological changes were observed, indicating that pigs have low susceptibility to HPAI A(H5N1), although they are perfect mixing vessels for avian, swine, and seasonal human IAV [83]. Another study in pigs using the mink-derived clade 2.3.4.4b H5N1 virus isolate, inoculated via intranasal, oral, and intratracheal routes, demonstrated fever and lethargy in inoculated pigs. However, the pigs regained normalcy 2 days post-infection and showed no clinical signs for the 3 weeks of the study. Respiratory tissue tropism was observed in pigs with moderate to severe pneumonic pathologic changes at 3 days post-challenge. However, neuropathological lesions were not present. The hallmark mammalian adaptive mutations PB2-E627K and PB2-E627V were observed in oropharyngeal samples [84].

Similarly, a study performed in ferrets showed that HPAI A/Indonesia/5/2005 (H5N1) can enter the CNS through the olfactory nerve and replicate effectively [85]. Viruses may acquire substitutions that enable adaptation to the CNS, and substantial virus production in the brain can result in severe meningoencephalitis in ferrets. The intraocular infection of H5N1 A/Chile/25945/2023 clade 2.3.4.4b in ferrets also caused fatal infection and facilitated contact transmission [86]. Interestingly, aerosol transmission was not observed in ferrets infected with bovine H5N1 [82]. Another study in ferrets using mink-derived H5N1 clade 2.3.4.4b also resulted in fatal infections and showed transmission by contact and aerosol to 75% and 37.5% of ferrets, respectively [87]. A study on a mouse model suggested that PB2-627K enables the virus to replicate efficiently in the mammals’ upper and lower respiratory tracts [88].

## 3. Neuropathophysiology of H5Nx Infections in Mammals

Unlike LPAIV or pandemic or seasonal viruses, HPAI H5Nx viruses affect the brain, with neurological disease being the main clinical manifestation in birds and mammalian hosts, including humans. In most cases, respiratory symptoms are either rare or absent in these infections. As described earlier, neurological disease has been reported in marine and domestic/companion animal hosts. The neuropathogenic potential of HPAI A(H5N1) Gs/Gd lineage is more pronounced than the H5N1 clade 2.3.4.4b [67]. The neuroinvasiveness of HPAI H5Nx viruses depends on the viral permissibility and spread through cranial nerves or through blood via the blood–brain barrier or blood CSF barrier, although hematogenous spread is not common in mammals [37]. In mammals, HPAI H5Nx viruses utilize the olfactory and trigeminal nerve in the nasal cavity, the facial and glossopharyngeal nerves in the upper respiratory tract, the vagus nerve in the lower respiratory tract, and the upper thoracic sympathetic nerves for CNS entry. The possible routes could be a combination of the digestive, respiratory, and olfactory routes [37].

HPAI H5Nx viruses attach to epithelial cells in the olfactory mucosa and can replicate efficiently in various cell types including sensory neurons. Upon entry into the olfactory sensory neurons, the virus can spread into the olfactory bulb and enter through the ethmoid cribriform plate to reach the synapses. The virus then spreads to glomeruli, periglomerular cells, and mitral cells and enters CSF through the fluid-filled areas lined by olfactory sheathing cells, ultimately reaching meninges. Similarly, the virus can also spread through the trigeminal and facial nerves (vestibulocochlear nerve), as demonstrated by the presence of viral antigens in the trigeminal ganglion or facial nucleus in mice and ferrets [37,85].

The neurotropism of the HPAI A(H5N1) virus depends on the HA binding specificity to the receptor. All the different types of cells in the CNS, such as neurons, glial cells, neuro endothelial cells, choroid plexus cells, and pericytes are permissive to the HPAI H5Nx viruses, as shown by the immunohistochemistry data [37,64]. Although not well characterized, previous studies have shown that CNS possesses both α(2,3)-sialic acids and α(2,6)-sialic acids in different cell types. Further, significant neuropathological changes observed in the brain and the presence of viral antigens in the CNS cells of different mammalian hosts indicate that neuroinvasion and neuropathogenesis are typical features of HPAI A(H5N1) viruses [37].

## 4. HPAI A(H5N1) Virus Infection in Humans

According to the World Health Organization (WHO), 873 people have been infected with the H5N1 virus worldwide since 2003, and 458 of them have lost their lives [89]. Human patients infected with H5N1 can develop respiratory symptoms ranging from asymptotic or mild to severe respiratory symptoms, including pneumonia. Besides respiratory symptoms, gastrointestinal symptoms such as diarrhea were also frequently reported in patients. In a fatal case of H5N1 virus infection in a child in southern Vietnam, the patient showed severe diarrhea but no respiratory symptoms. Further investigation demonstrated that the virus infection resulted in acute encephalitis in the patient [90].

In April 2024, a dairy farm worker in Texas tested positive for HPAI A(H5N1) virus after exposure to H5N1-infected dairy cows [91,92]. So far, eight human cases have been linked to bovine H5N1 (https://www.cdc.gov/bird-flu/h5-monitoring/index.html, accessed on 14 October 2024). Altogether, 14 total human cases have been reported since 2022, mostly in dairy farm workers in the US. The symptoms reported are mild conjunctivitis, ocular discharge, and infrequent cold symptoms. The first H5N1 human infection case was reported in 2022 in Colorado. The patient who had direct exposure to H5N1 virus-infected poultry exhibited fatigue as the only symptom [93]. Conjunctivitis is a typical symptom in humans infected with HPAI viruses. Fouchier et al. reported that 78 of 86 H7N7 virus-infected patients showed conjunctivitis [94]. The conjunctival route can serve as an entry portal for HPAI viruses, as the ocular surface harbors alpha 2,3 linked sialic acid (avian-type receptor) [95]. Therefore, avian influenza viruses can bind to these alpha 2,3 linked sialic acids and initiate the infection in the eyes.

It is worth noting that the human specimen collected in 2024 from an H5N1-positive patient in Texas was closely related to the HPAI A(H5N1) isolated from dairy cattle. The genotype was B3.13 with PA, HA, NA, and M genes from the Eurasian lineage and PB2, PB1, NP, and NS from the American lineage [96]. Interestingly, the human-derived H5N1 harbors a substitution (E627K) in PB2 that was not present in Texas dairy cattle and birds. This PB2 E627K substitution may be an acquired mutation during replication in that patient [96]. This substitution can enhance viral polymerase activity in human and other mammalian cells and is considered an important mammalian-adapting substitution [97].

On 29 March 2023, a 53-year-old man was reported to be infected with the HPAI A(H5N1) virus, and genomic analysis detected a D701N substitution in the virus PB2 segment [98]. The PB2-D701N mutation also has been shown to enhance viral polymerase activity in human cells, as determined in a luciferase-minigene replication assay [99]. In addition to E627K and D701N in PB2, other substitutions such as T271A [60,100] may also increase viral polymerase activity in mammalian cells and are important for virus transmissibility in mammals including humans. The increased polymerase activity may facilitate HPAI virus replication in new hosts. Of note, these adaptation substitutions can facilitate virus replication in mammals, but there is no clear evidence that these substitutions contribute to the transmission among mammals.

## 5. HA, NA, and NS Also Determine Virus Tropism and Host Range

Viral hemagglutinin (HA) binds to sialic acid (Sia) receptors on the cell surface to initiate the infection process, and substitutions in HA can alter the receptor binding affinity, which affects viral replication, transmission, and host range. Influenza viruses circulating in avian species usually bind to alpha-2,3 linkage Sia receptors whereas viruses in humans usually bind to alpha-2,6 linkage. Substitutions in HA may change the virus preference of Sia receptor linkage, which is demonstrated by the virus strain that caused the human pandemic in 1968. Evolutionary studies using the H3 HA sequences from avian, human, and other non-human mammalian hosts have shown that the avian virus can infect humans after acquiring six amino acid substitutions in the HA [101]. The loss of glycosylation at HA position 158 can increase alpha-2,6 sialic acid receptor binding affinity. Gao et al. reported that the T160A substitution in HA, which results in the loss of the glycosylation site at HA-158-160, can affect the viral pathogenicity in mice [102]. Furthermore, Jang et al. demonstrated that another substitution in H5 HA, N193D, can enhance the binding to alpha-2,6 linage [103]. The H5 adaptation in human hosts depends on the mutations that promote receptor specificity and better fusion of the viral membrane facilitating replication in the upper respiratory tract. The avian influenza viruses, including the H5 subtype, mediate fusion at a high pH compared to the human influenza viruses, so any HA-specific mutations that can lower the fusion pH could enhance virus stability in the acidic pH of the human upper respiratory tract, thus improving replication and transmissibility [104].

Neuraminidase (NA) is another glycoprotein on the virus surface, and it acts as a sialidase enzyme to cleave sialic acid from the host cell surface and facilitate the release of newly assembled progeny virions from infected cells. NA possesses a second sialic acid-binding site (2SBS) that is important for HA-NA balance and virus replication [105]. Of note, 2SBS is highly conserved in avian IAVs, whereas it is absent in human viruses. Additionally, 2SBS binds to alpha-2,3 linked Sia receptors, but substitution in 2SBS can reduce the binding to alpha-2,3 linked Sia receptors and may enhance the binding to alpha-2,6 linked Sia receptors. The HPAI A(H5N1) virus responsible for numerous deaths of farmed mink has disrupted 2SBS [106]. The loss of 2SBS results in enhanced binding to human-type receptors; therefore, 2SBS serves as an important determinant of the virus host range.

The non-structural protein 1 (NS1) has been shown to be crucial for virulence, pathogenicity, and host range [107,108]. NS1 can enhance the expression level of cytokines and limit the induction of interferon (IFN)-beta by multiple pathways, thereby inhibiting the host antiviral responses. The deletion of NS1 gene in the influenza A virus (A/PR/8/34) results in reduced replication in embryonated chicken eggs because of the IFN-mediated antiviral effects [109]. A recombinant H7N1 HPAI virus with the NS segment replaced by the H5N1 virus NS replicates more efficiently in human cell lines and induces stronger pathogenicity in chicken embryos compared to the wild-type virus [60]. In another study, a recombinant H1N1 virus with the H5N1 NS gene conferred more prolonged clinical manifestations in pigs compared to the wild-type virus. This effect was conferred by a D92E substitution in NS1 [110]. The amino acid at position 149 of NS1 has also been reported to impact the pathogenicity of the H5N1 virus in chickens. A recombinant H5N1 virus A/goose/Guangdong/1/96 with Ala at position 149 of NS1 can limit the interferon protein expression in chicken embryo fibroblasts, whereas a virus with Val at position 149 cannot [110]. More studies are needed to investigate the NS1 determinants of adaptation to mammals.

## 6. Vaccines Against HPAI A(H5N1)

The reverse genetics approach provides a novel and important way to investigate virus gene functions, virus virulence, transmission, and antivirals [10,111,112,113,114,115,116,117]. Moreover, the reverse genetics approach is also critical to the rapid generation of vaccine strains [118,119,120]. Reverse genetics-based vaccines were developed and demonstrated high protective efficacy in influenza viruses, including HPAI A(H5N1) viruses [118,120,121,122,123,124]. Webby et al. used a reverse genetics approach to generate a candidate reference virus strain in response to a pandemic alert issued by WHO in 2003 [120].

Currently, commercial vaccines against H5N1 for poultry are not used in the US. poultry. The HPAI A(H5N1)-affected chickens were culled to eradicate the viruses and their poultry reservoir. However, vaccines were used to protect the endangered California condor from HPAI A(H5N1) infections, making it the first instance that the US vaccinated any endangered bird species to curb HPAI virus infections [125]. Vaccinations against HPAI A(H5N1) are common in China, Egypt, Indonesia, and Vietnam [126,127,128,129]. Due to the virus’s rapid evolution, vaccine strains need to be updated in a timely manner to maintain vaccine protective efficacy [130]. Vaccines against HPAI A(H5N1) viruses were shown to prevent infections in chickens, geese, and ducks, and these vaccines can reduce virus replication in gastrointestinal and respiratory tracts [131]. The implementation of vaccines against HPAI A(H5N1) in poultry farms, and the timely culling of the infected flock during outbreaks, can reduce the chance of human and wild bird exposure to the HPAI virus [126]. Poultry vaccination can help control HPAI in wild birds, as vaccine-mediated protective immune responses can reduce viral shedding in the environment. USDA has worked on the evaluation of the efficacy of four vaccine candidates against HPAI in poultry [132].

There is growing concern that the adaptation of the HPAI A(H5N1) virus to dairy cattle may result in human-to-human transmission. Seasonal influenza vaccines cannot provide any protection against H5N1 virus infections, highlighting the need for a vaccine against H5N1 infections in humans. Although no mutations linked to human-to-human transmission were detected in the virus sequences, the pandemic potential of the H5N1 virus cannot be overlooked, given the high mutation rate that can readily occur and accumulate during virus replication over time. As part of pandemic preparedness, the Centers for Disease Control and Prevention (CDC) has developed an H5 candidate vaccine virus (CVV) that can be used for the production of human vaccines if needed [133]. It should be noted that vaccines against HPAI A(H5N1) viruses need to be updated due to the antigenic drift or shift associated with influenza virus mobility. Therefore, ongoing virus surveillance and antigenic analysis are crucial for selecting candidate vaccine strains and maintaining optimal vaccine efficacy.

## 7. Conclusions and Future Directions

The sudden emergence of HPAI A(H5N1) clade 2.3.4.4b viruses in dairy cows and its detection in milk samples across multiple US states raise several immediate questions that need to be addressed in future research.

First, among influenza A subtypes, is H5N1 clade 2.3.4.4b only a group of the virus capable of jumping to dairy cows from birds and causing sustainable cow-to-cow transmission? Several studies published in the late 1990s have provided good evidence that other subtypes of influenza A virus such as H3 could infect dairy cows [40] and cause a drop in milk production and other symptoms similar to those observed in the US dairy cows infected with HPAI A(H5N1) virus. In this regard, the infection of dairy cows by an influenza A virus is not entirely new. The uniqueness of this influenza endemic in cattle lies in the BSL3 agent HPAI A(H5N1), which has the potential to cause a human pandemic if human adaptation mutations are acquired. A comprehensive investigation of other subtypes of IAV in addition to H5N1 is needed to determine their ability to infect dairy cows and impact milk production. Identifying potential differences between different strains or subtypes may lay the foundation for future research toward identifying cattle adaptation mutations.

Second, despite the mammary gland being one of the established in vivo targets for H5N1 clade 2.3.4.4b, the virus was detected in nasal shedding as well as in the lungs of infected cattle. Asymptomatic cows appear to be responsible for the widespread nature of this disease in US dairy cow farms. It has also been shown that the virus was detected in the lungs of clinically normal cows. It can be envisioned that the H5N1 clade 2.3.4.4b has a very broad tropism ranging from the respiratory and gastrointestinal tracts to the mammary gland tissues, which warrants further investigation. Also, it remains unclear whether beef cattle breeds and heifers are susceptible to HPAI A(H5N1) infection. More work is needed to define tissue tropism and the host range of this emerging virus in cattle.

Third, some gathered evidence suggests that milk and/or contaminated equipment on dairy cow farms play an important role in cow-to-cow transmission. Whether the major mode of transmission occurs through the fecal-oral route or through aerosol droplets during the milk collection process is not well known. Considering the virus detection in nasal shedding and lung tissues, there is a need to address the transmission through the classical respiratory tract. A significant amount of viral load is found in milk, indicating that the mammary gland is a primary target organ where HPAI A(H5N1) replicates. Whether the virus reaches the mammary gland through the blood–milk barrier (systemic infection and viremia) or a local route warrants future investigation. Also, it will be interesting to know whether bovine-origin H5N1 exhibits any neurotropism in cows, as brain and tissue samples from the small ruminants tested positive for H5N1.

Finally, bovine immunity, especially antibody responses to influenza virus infection, has not been well-studied in the past due to a long-held belief that cattle are not susceptible hosts to influenza A viruses. Several studies on bovine influenza D viruses showed that persistent infections frequently occur in bovine herds, and preexisting immunity is somehow short-lived [134]. Investigations of the durability and potency of protective antibody responses against HPAI A(H5N1) in dairy cows should address this important question, which will provide insights into the successful implementation of vaccine strategies, which are critical to the control and prevention of future influenza epidemics in the cattle industry.
